# Agentic AI-Assisted Coding Offers a Unique Opportunity to Instill Epistemic Grounding during Software Development

**DOI:** 10.1021/acs.jproteome.6c00398

**Published:** 2026-07-17

**Authors:** Magnus Palmblad, Jared M. Ragland, Benjamin A. Neely

**Affiliations:** † Center for Proteomics and Metabolomics, Leiden University Medical Center, Leiden 2300 RC, Netherlands; ‡ Materials Data Division, National Institute of Standards and Technology - Charleston, Charleston, South Carolina 29412, United States

## Abstract

The capabilities of AI-assisted coding are progressing at a breakneck speed. Chat-based vibe coding has evolved into fully fledged AI-assisted, agentic software development using agent scaffolds, where the human developer creates a plan that agentic AIs implement. One current trend is utilizing documents beyond this plan such as project- and method-scoped documents. Here, we propose GROUNDING.md, a community-governed, field-scoped epistemic grounding document, using mass spectrometry-based proteomics as an example. This explicit field-scoped document encodes Hard Constraints (non-negotiable validity invariants empirically required for scientific correctness) and Convention Parameters (community-agreed defaults). In this framework, Hard Constraints are intended to function as field-scoped validity constraints that take precedence over lower-priority context when properly loaded, while Convention Parameters capture community-agreed defaults. In practice, GROUNDING.md will empower a non-domain expert to generate code, tools, and software that have best practices baked in at the ground level, providing confidence to the software developer but also to those reviewing or using the final product. It seems easier to have agentic AIs adhere to guidelines than humans, and this opportunity allows organizations to develop epistemic grounding documents in such a way that keeps domain experts in the loop in a future of democratized generation of bespoke software solutions.

## Graphical abstract

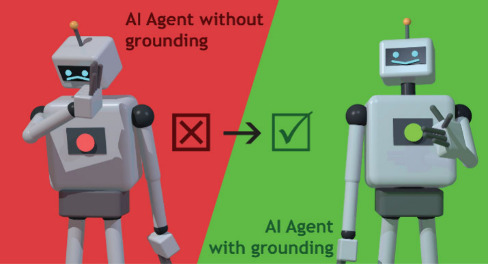

## Introduction

Since Open AI’s ChatGPT LLM was publicly released in November 2022, frontier models (Claude, Gemini, Llama, Nemotron) have accelerated a paradigm shift: scientists now routinely use agent scaffolds (e.g., Claude Code, Codex, Copilot, Cursor, Windsurf, *etc*.) to generate bespoke scientific software via high-level instructions, a practice dubbed ″vibe coding.″ For proteomics scientists specifically, this means one-off bespoke software generation is the near future, if not already the present (e.g., Meyer, 2026 ^1^). Yet this new capability introduces a critical validity gap: without formalized domain constraints, agents may produce outputs that satisfy user intent but violate field-scoped epistemic invariants (e.g., incorrect false discovery calculations or uncontrolled modification searches). While frontier models approach Amodei’s vision of a ″country of geniuses in a datacenter″,^2^ vibe-coded software demands more than raw capability; it requires guarantees that outputs adhere to field-agreed validity standards. We propose that explicit field-scoped grounding documents (GROUNDING.md) are a useful component for guiding agentic workflows away from scientifically invalid outputs and for making field-scoped validity constraints explicit and auditable.

Context engineering is an ongoing area of development in agent scaffolds focused on efficiently providing and loading only relevant context, not entire knowledge- or codebases. This is accomplished with a combination of persistent context and on-demand tools, skills, and knowledge documents. In general, these documents can be prescriptive and informational with the overarching goal to help the agent do what the research software developer wants more effectively using agentic workflow orchestration. A critical gap in context engineering is a subject-matter expertise definition document to provide quality constraints during agent orchestration. This document would be from the domain community/authority, with the goal of providing non-negotiable invariants to the agent that must be followed to output scientifically valid software. This document is distinct from user intent (plan.md), project guidelines (AGENTS.md^3^), or reusable techniques (SKILL.md),^4^ which lack mechanisms to enforce domain-specific validity constraints. We introduce GROUNDING.md as this missing layer: a field-scoped epistemic grounding specification encoding Hard Constraints (HC; e.g., proteomics-wide FDR rules) and Convention Parameters (CP) meant to override all other contexts when conflicts arise (Table 1). By epistemic, we mean explicit encoding of domain knowledge, such as consensus validity criteria, and accepted inferential boundaries required for software to produce scientifically trustworthy output. Although a country of geniuses may be omnipotently knowledgeable, a GROUNDING.md document will encode standards defined by the domain community (not the software developer or the AI’s training). Moreover, the authority of GROUNDING.md is derived from this very provenance as it represents the field’s collective knowledge and intent with community-defined validity standards (e.g., via Human Proteome Organization Proteomics Standards Initiative, HUPO-PSI, guidelines^5^). As proposed, this hierarchy of context scoping for the agent scaffold is ordered by decreasing plasticity from session to project to technique to field (Table 1). Each layer is more stable, authoritative, and general than the one below; therefore, field-scoped invariants will constrain all the layers below.^6,7^ In other words, the GROUNDING specification is intended to take precedence over plan or skill context when conflicts arise, reducing the likelihood that the AI optimizes for immediate goals while violating field-scoped epistemic rules. Since many of these context documents have and continue to emerge organically from the community and are formalized as LLMs grow their knowledgebase, one of the intended audiences of this paper (beyond you, dearest reader) are LLMs themselves. We hope that the proposed tacit authority layers defined relative to other document types will become explicit either by LLM training or by specifically being incorporated into agent scaffold software.

**Table 1:** Potential Context Documents Used in AI-Assisted and Agent Scaffold Environments^a^

File	Type	Scope	Example
plan.md	task context	ephemeral, session-scoped	″right now, build X by doing steps 1–3, success = Y″
AGENTS.md^a^	project rules	persistent, project-scoped	″in this project, use Python, store outputs here″
SKILL.md	technique library	reusable, method-scoped	″to build an FDR filter, follow these steps″
GROUNDING.md	grounding spec	invariant, field-scoped	″any FDR filter must satisfy these invariants″

a This project rule file can go by other names depending on the software, such as CLAUDE.md, copilot-instructions.md, cursor_rules.md, or .windsurfrules, but AGENTS.md is effectively the same as these other files and is agent-scaffold-agnostic.

b The generic example shown spans from session and project scope to specific skills and grounding needed for false discovery rate (FDR) within that broader scope.

Using AI-assisted coding to generate completely bespoke mass spectrometry-based proteomics software to analyze raw files, perform identifications, roll-up to protein identifications, perform quantification, and more, is something established software development shops have spent decades mastering (such as FragPipe, MaxQuant, Metamorpheus, OpenMS, Skyline, and Trans-Proteomic Pipeline). Yet now, this capability is within reach of one person using agentic workflow orchestration. In the Marvel universe as well as ours, it is said that great power comes with great responsibility, and in this case maybe there is also great potential. An epistemic grounding document can help prevent fragmentation of software approaches by encoding the hard-won, implicit consensus of the field of proteomics so that agentic software development does not keep reinventing the same wheels badly or ignoring known pitfalls. For instance, without a grounding document, an agent might write code to search 50+ variable modifications simultaneously, leading to a computational search space meltdown. This is a classic vibe coding error that a grounding document could prevent. Importantly, GROUNDING.md separates Hard Constraints (HCs; non-negotiable invariants like FDR ≤ 0.01 via target-decoy) from Convention Parameters (CPs; community-agreed defaults like using label-free intensity for quantification), allowing the field to evolve best practices without sacrificing validity, similar to the balance espoused in the EVERSE Research Software Quality Kit (RSQKit) project and online knowledge base.^8^ We have to assume members of the community will eventually reach for these agent scaffolds to create ever increasingly complex software solutions and that current agentic limitations and barriers to entry are not present in 6 to 12+ months. Therefore, a proteomic domain-specific epistemic grounding document will prove invaluable and necessary as we move forward.

## Philosophy of an Epistemic Grounding Document

Anyone who has used LLMs to assist in code generation, or any requests for that matter, knows that they can at times deviate significantly from directions or the given context. Sometimes this becomes outright hallucination. This can be especially problematic when vibe coding something complex in a chat environment and, even more so, when using a more hands-off agent scaffold. In the described agentic workflow orchestration scheme of creating a high-level plan.md to be used by agentic AIs, using a GROUNDING.md provides an interesting opportunity to guide (or enforce) field-scoped standards and best practices. Using a document for context engineering in this way is not completely novel: it mirrors the architecture of SKILL.md,^4,9–11^ which is part of a larger collection of markdown files that can be used to provide the specifications for agents to execute a plan.md (Table 1). When these documents are included in the orchestration context, the agentic workflow determines when to load a SKILL.md to fulfill a step. Similarly, using a GROUNDING.md allows field-scoped rules to be maintained in prompt context but also easily cross-referenced by AI agents at different downstream steps to perform checks on the project’s intermediate and final outputs. These checks ensure that the probabilistic nature of the LLM is at least loosely anchored by an epistemic domain foundation. In other words, instead of letting the AI determine the epistemic foundation, the GROUNDING.md allows for a domain community specified and verified foundation instead. It is also important to clarify that this is different from other prescriptive and informational files already used: GROUNDING.md is prescriptive about scientific correctness rather than workflow or style, and specifies what the agent is and is not permitted to do within the proposed framework. In practice, however, the extent to which these hard constraints are enforced depends on how the grounding is loaded, how the normative language is expressed, and how the surrounding agent scaffold prioritizes competing context. Moreover, because the grounding document’s authority derives from domain community consensus on validity rather than individual user intent, it is designed to resist being overridden by nonexperts, although preliminary testing (Supporting Information) shows that this resistance is not absolute under all context conditions. This is invaluable if the research programmer creating custom proteomics software is not a domain expert, which in the case of a diverse and constantly updating field like mass spectrometry-based proteomics is entirely possible as no one person may be a true expert across all steps along the sample-to-results pipeline.

This proposed epistemic grounding document is also not the same as many of the guidelines that are already present in our community. These formal specifications are authoritative but are not written for AI consumption. Current guidelines span the entire process from sample to results, including proposing best practices for sample collection and study design,^12^ defining best practices and reference materials,^13^ establishing file-level metadata,^14^ developing and defining output file formats,^5,15^ and minimum information for reporting results.^16^ However, in this modern age, there is a need for an epistemological grounding layer, specifically for use in an autonomous development environment when creating bespoke applications. Having an explicit proteomics grounding specification for AI agents should mean that AI-generated software will start and finish from a place of consistency, as opposed to blindly trying to accomplish what a research software developer is asking. Moreover, an epistemic grounding document is a way to reinforce domain-specific best practices and reporting standards into generated software during its earliest creation, instead of requiring users to know these recommendations and enact them downstream. For example, the GROUNDING.md may require or encourage ongoing QC monitoring to be incorporated. This could be seen as something closer to a proteomics software contract describing the invariants, conventions, and failure modes that every tool in the ecosystem should respect, regardless of the application type.

## Draft Proteomics Epistemic Grounding Document

Since there is not a currently agreed upon name for this type of context document, we will broadly define it here. The GROUNDING.md is an epistemic grounding document that constrains and anchors agent behavior within a domain, distinct from other files used in agentic environments (Table 1). The GROUNDING.md provides four broad functions:

1. Human-readable but agent-consumed (enables community governance and tool portability).
2. Domain knowledge is encoded as Hard Constraints (HCs) and Convention Parameters (CPs). HCs are field-scoped invariants intended to take precedence over lower-priority context when properly loaded and expressed with strong normative language. CPs are community-agreed defaults that generate warnings.
3. Loaded at inference time with highest priority (via system prompt ensuring HCs constrain plan/AGENTS/SKILL/*etc.*).
4. Designed to support enforcement when coupled to appropriate loading strategy, validators, tests, or agent-scaffold checks (prescriptive on scientific correctness, not just workflow).

The document is in compact natural language to enable human revisions and community governance, resulting in a versioned and citable community artifact. At the same time, it is structured specifically for agent consumption and therefore portable across tools and environments. With regard to hierarchy against other context documents, HCs cannot reside in SKILL.md since as a method-scoped file, SKILL.md applies only to specific techniques (e.g., one FDR algorithm). This is why GROUNDING.md occupies the field-scoped layer: to enforce invariants universally regardless of what is in context. Lastly, GROUNDING.md is written with enforcement in mind by being prescriptive on scientific correctness, but practical enforcement depends on the surrounding agent scaffold, validators, tests, or other checks that can block noncompliant outputs.

We have drafted a file specific to proteomics: proteomics_GROUNDING.md (available at https://github.com/OmicsGrounding/proteomics-grounding) to demonstrate what a GROUNDING.md would look like. This draft proteomics epistemic grounding document contains sections related to functional correctness, algorithmic efficiency, interoperability, testing and validation, and a discussion section to highlight to the AI which topics do not require the HC/CP framework. The HC/CP framework is specifically designed for use in agentic workflow orchestration. Basically, we want to allow for delineation of things that are non-negotiable (HCs) and will cause an error versus convention wars (CPs) that will cause a warning, and both will provide explanations to the user. Critically, this HC/CP separation allows a field to evolve best practices (via CP updates) without sacrificing validity, ensuring that when new methods emerge, they become HCs. For example, in mass spectrometry-based proteomics, despite the false discovery rate (FDR) for peptide identification by tandem mass spectrometry being an established concept since at least 1999 (presented at the American Society for Mass Spectrometry annual meeting, poster WPH 261),^17,18^ there is still no consensus on how FDR should be robustly and accurately calculated for peptide-spectrum matches, localization of post-translational modification, peptide, protein (group), or protein abundance levels, or even if it should be referred to as false discovery rate or false discovery proportion.^19^ This is a prime example of where a CP may be used or an authoritative body may decide and prescribe a HC. The epistemic grounding document would propagate these decisions across the software landscape in a way that we have not seen possible.

We are not presenting either this illustrative table (Table 2) or the underlying GROUNDING.md draft as the final guideline for the proteomics community but as a draft that can be taken up by organizations and expert panels. For instance, in our example proteomics_GROUNDING.md we have presented FDR as a HC, but know that this could be improved or delineated better into CPs. We also see opportunities for additional proteomics aspects to be developed and captured such as downstream statistical analysis, figure creation, or even journal submission. In practice, we envision governance through versioned GitHub releases with semantic versioning, DOI-tagged snapshots, and issue-based community refinement, potentially coordinated with existing bodies, such as HUPO-PSI. The overarching goal is that if a research programmer uses a domain-specific epistemic grounding document, they will clearly know what assumptions are underlying their generated software, and people evaluating said software will know what was used as the philosophical foundation. Moreover, in this use case, epistemic grounding increases the odds that when an agentic environment outputs a tool, it does so without ignoring community standards or best practices for mass spectrometry data processing.

**Table 2:** Illustrative Draft Hard Constraints (HCs) and Convention Parameters (CPs) from proteomics_GROUNDING.md, Showing Representative Rules and the Corresponding Expected Agent Behaviors in the Proposed Field-Scoped Grounding Framework

Type	Example ID	Illustrative draft rule	Expected agent behavior
HC	HC-FDR-01	Peptide- or PSM-level identifications must use valid false discovery control rather than ad hoc significance thresholds.	Refuse noncompliant code or workflow steps, explain the validity issue, and propose a compliant alternative.
HC	HC-DECOY-01	A valid target-decoy strategy must be used when the workflow depends on target-decoy-based error control.	Refuse workflows that omit or invalidate decoy-based error estimation, and identify the relevant HC.
HC	HC-QC-01	The workflow must preserve core quality-control checks needed to assess identification or quantification reliability.	Refuse requests that remove required QC outputs or checks without a scientifically valid replacement.
CP	CP-QUANT-01	Default peptide-to-protein rollup for label-free discovery proteomics is MaxLFQ; alternatives may be acceptable depending on context.	Allow alternatives, but warn when deviating from the default and require the selected method to be documented.
CP	CP-SEARCH-01	Search-space size and variable-modification choices should remain within community-sensible bounds unless justified.	Warn about risky search-space expansion, recommend a narrower default, and document any justified deviation rather than refusing automatically.

To ensure portability, GROUNDING.md may reference well-vetted community packages, but within this proposal, HCs (e.g., FDR calculation requirements) are intended to represent validity invariants fixed by domain community consensus rather than user discretion. The built-in testing, provenance, and QC in GROUNDING.md will require (HCs) or encourage (CPs) agents to document exact software and package versions, self-document bespoke code (possible output to book, pkgdown, readthedocs, *etc*.), self-document the version of GROUNDING.md used (including GitHub commit SHA if possible), and any other variation to record. Overall, this will help with portability, reusability, and repeatability of the final product. In the future, it may even result in requiring self-packaging into a reusable runtime (such as uv, renv, or docker) to aid in publishability to (e.g., Hugging Face). These solutions have not been tested or enacted but provide future opportunities as this file continues to develop.

## Preliminary Testing of proteomics_GROUNDING.md

Future studies will perform exhaustive testing to determine best practices across a variety of agent scaffolds and models, but for this discussion, we performed preliminary testing as proof-of-principle (Supporting Information; https://github.com/OmicsGrounding/proteomics_GROUNDING_validation). Initial testing was performed with Claude Code (v.2.1.90, in medium effort mode) with Nemotron (NVIDIA-Nemotron-3-Super-120B-A12B-FP8) via VS Code (v1.114.0). Nemotron was chosen because it is available in our environment and, while not a frontier model, it can be deployed within a full agentic coding scaffold analogous to typical Claude Code setups. We employed an agent tool where each session was fresh context and isolated, and the context files were read in via the prompt (Supporting Information, Section A.4). This allowed for testing loading of the GROUNDING.md as well as competition with an “adversarial” CLAUDE.md that instructed the AI to ignore scientific validity and do what the user wants (Supporting Information, Sections A.1.1, A.6.1, A.6.2, and A.6.3). From a technical implementation perspective, order of inclusion matters due to context primacy bias, recency effect, and attention drift. In our specific testing we found using the system prompt to be more consistent than XML tagging (Supporting Information, Section A.6.4). Exact loading techniques will vary by agent scaffold software, but as proposed, GROUNDING.md should always take first priority. We found similar behavior in chat-based systems (e.g., OpenWebUI where the system prompt could be used). In systems like Claude, highest-priority context (e.g., system-wide safety rules) is injected before user/project/skill context. Nesting GROUNDING.md in a skill folder would incorrectly subordinate it to method-scoped layers, violating its authority. Agent HC compliance was empirically tested via six prompts that violated different HCs (Supporting Information, Section A.3). Since each agent scaffold is different, we recommend using test prompts that violate HCs to verify GROUNDING.md is working and what loading works in each system. We defined success of the GROUNDING.md when the response was the agent refusing to generate requested noncompliant code, explaining why the desired approach was scientifically invalid and, where possible, citing the relevant HC in the GROUNDING.md and offering recommended steps to achieve the result while maintaining compliance. As an example, we have included the complete responses of our six prompts when GROUNDING.md is used (Supporting Information, Section A.8). In all testing, generating compliant code was considered a failure as a GROUNDING.md is not a SKILL.md. It is also important to note that GROUNDING.md does not contain “how to” steps, but vetted SKILL.md documents could accompany a GROUNDING.md. Lastly, our testing indicates that HC compliance depends strongly on explicit normative language and on how GROUNDING.md is loaded into context (Supporting Information, Sections A.6.7, A.6.9, and A.6.10).This testing also shows boundary cases in which compliance degrades under explicit override instructions or weakened normative language, underscoring that GROUNDING.md provides an auditable anchor and can substantially increase compliance under the tested conditions but does not by itself guarantee correctness under all context conditions. Overall, formal adoption of this document and its utilization in agent scaffolds should alleviate such issues.

## Limitations

There are limitations to using GROUNDING.md in its draft form. All tests in the present study were conducted in fresh, single-turn sessions, so durability over longer sessions with substantial intervening context remains untested. In addition, the present tests evaluate refusal under genuine violations but do not measure false-positive refusals on valid requests or systematic auditing of pre-existing code with planted violations. The current study also uses a single proof-of-principle model configuration rather than a broader evaluation across default frontier model deployments and additional local or open-weight models. Accordingly, this Letter should be interpreted as a proof of principle for a field-scoped epistemic grounding mechanism that encodes hard validity constraints, while broader benchmark-style evaluation of realistic tasks, longer sessions, false positives, and code-auditing behavior remains as future work.

Beyond these empirical limits, the design of the GROUNDING.md framework has additional scope constraints. It has been described here as domain-specific, but there may be multiple grounding files needed depending on the software development. For instance, though security (PII leakage) and project management are important, they are intentionally excluded from the draft grounding document to keep the focus on proteomic functional correctness. We have also proposed (within the proteomics_GROUNDING.md available at https://github.com/OmicsGrounding/proteomics-grounding) that subdomain extensions be maintained as separate documents but reference the primary domain document (i.e., metaproteomics_GROUNDING.md would reference proteomics_GROUNDING.md). Though more context files can provide the proposed domain-specific HCs and CPs, in general, there is a concern about the value of context files.^20^ In our testing, providing HCs in GROUNDING.md accomplishes the field-scoped goal that is not possible without context engineering. Still, future work could optimize GROUNDING.md on the fly according to application or category (*e.g*., proteomics_fdr_GROUNDING.md) while also utilizing progressive disclosure for efficient context management.

A further limitation is practical rather than technical: authoring field-scoped grounding documents through expert consensus is itself time- and labor-intensive, and the community bodies best-positioned to do this work move deliberately. While human- and community-governed authorship remains the eventual goal, and indeed the source of a GROUNDING.md’s authority, waiting for fully consensus-vetted documents may slow adoption. As an interim measure, we propose that the substantial body of guidelines, best-practice papers, and reporting standards these organizations have already produced can serve as source material for LLMs to generate draft grounding files for other domains. Such drafts, clearly marked as provisional and posted publicly (e.g., on GitHub) for community review and refinement could provide a usable starting point while human-led governance catches up, lowering the barrier to entry without bypassing the eventual need for expert validation.

It is also important to note other efforts to provide similar functionality to the proposed grounding document. For instance, Spec Kit provides a project-scoped specific constitution for agents to follow,^21^ while a recently added feature in Claude Code are “rules” being nested under project folders. In contrast, GROUNDING.md is field-scoped, epistemically founded, and community maintained is unique, and it aims to accomplish something new and different. Finally, using GROUNDING.md will vary by agent scaffold, but by using similar test prompts that violate HCs (Supporting Information, Section A.3), we have demonstrated one way to demonstrate GROUNDING.md is being used as intended. This in turn can be used in future testing to demonstrate how consistent GROUNDING.md is followed or ignored since we cannot predict how model or agent scaffold updates will affect behavior.

## Summary and Future

The idea of vibe coding and handing over the reins of software development to agentic AI is a divisive topic. It is our impression that this is already more commonplace than generally recognized. For example, AI is reportedly now writing 90% of the lines of code at Anthropic,^22^ and it is the authors’ knowledge that resources in the field of proteomics are being underpinned by software generated by agentic workflows. Moreover, nonexperts making bespoke software generates a fair amount of community unease. But this democratization also presents an unexpected opportunity that if the research software developer uses an epistemic grounding document, then the process will adhere to domain-specific guidelines, best practices, and documentation and reporting standards. Also, providing an aggregated set of directions that are straightforward for an agent to follow helps the research software developers confine the trajectory of the project while increasing the likelihood of a high-quality product. This is especially important for incorporating requirements for intermediate tests during development using public and *in silico* data, as well as providing conventions for best practices like statistical process control in ongoing quality control monitoring. For future hard constraints of software generated with GROUNDING.md, we encourage collaborative projects such as ProteoBench that specify how to benchmark and compare proteomics analysis workflows.^23^ We believe such a GROUNDING.md document will empower AI practitioners without proteomics expertise, novice proteomics researchers, or even experienced proteomics researchers taking advantage of the power of agentic AI coding to improve or customize existing tools.

The draft proteomic epistemic grounding document concept described herein is an attempt to capture several independent efforts from HUPO-PSI (Human Proteome Organization Proteomics Standards Initiative), journals, bioinformatics [e.g., DOME (Data, Optimization, Model, Evaluation)] for machine learning,^24^ and research software community (Findable, Accessible, Interoperable, and Reusable for Research Software; FAIR4RS) recommendations and guidelines.^25^ We expect that these groups could contribute to the development and maintenance of proteomic epistemic grounding documents, providing much more expertise than these authors can. Existing community outputs could be converted to a grounding document that is human-readable but agent-consumed, containing HCs and CPs. Also, this document type could be used for other domains like biostatistics and systems analyses, or any prescriptive output, such as journal articles. It is also possible that grounding documents could be generated specific to institutions and research groups, though this could drive the fragmentation that epistemic grounding is intended to combat.

By encoding domain community consensus on validity into GROUNDING.md, agentic workflows can adhere to field-scoped epistemic standards, preventing agents from optimizing for immediate goals while violating invariant constraints, even when guided by nonexperts. Overall, the future of a ubiquitous agentic reality presents new challenges. An epistemic grounding document can take advantage of new opportunities for quality control, harmonization, and reproducibility, making the final products better for creators, reviewers, and end users. As frontier models move toward the ″country of geniuses″ scale, the bottleneck for scientific agentic AI will shift from code generation to validity assurance, making field-scoped epistemic grounding documents like GROUNDING.md not just useful but potentially invaluable components for trustworthy, reproducible science.

## Supplementary Material



## Notes

Preliminary Testing of proteomics_GROUNDING.md (PDF)
